# Xanthine oxidoreductase activity is correlated with hepatic steatosis

**DOI:** 10.1038/s41598-022-16688-0

**Published:** 2022-07-19

**Authors:** Chisako Yagi, Yoshiki Kusunoki, Taku Tsunoda, Takayo Murase, Takashi Nakamura, Keiko Osugi, Mana Ohigashi, Akiko Morimoto, Akio Miyoshi, Miki Kakutani-Hatayama, Kae Kosaka-Hamamoto, Manabu Kadoya, Kosuke Konishi, Takuhito Shoji, Hidenori Koyama

**Affiliations:** 1 Department of Diabetes, Endocrinology and Clinical Immunology, Hyogo Medical University, 1-1, Mukogawa-cho, Nishinomiya, Hyogo 663-8501 Japan; 2 Radioisotope and Chemical Analysis Center, Laboratory Management Department, Sanwa Kagaku Kenkyusho, Nagoya, Japan

**Keywords:** Endocrinology, Gastroenterology

## Abstract

The enzyme xanthine oxidoreductase (XOR) catalyzes the synthesis of uric acid (UA) from hypoxanthine and xanthine, which are products of purine metabolism starting from ribose-5-phosphate. Several studies suggested a relationship between hyperuricemia and hepatic steatosis; however, few previous studies have directly examined the relationship between XOR activity and hepatic steatosis. A total of 223 subjects with one or more cardiovascular risk factors were enrolled. The liver-to-spleen (L/S) ratio on computed tomography and the hepatic steatosis index (HSI) were used to assess hepatic steatosis. We used a newly developed highly sensitive assay based on [^13^C_2_, ^15^N_2_] xanthine and liquid chromatography/triple quadrupole mass spectrometry to measure plasma XOR activity. Subjects with the L/S ratio of < 1.1 and the HSI of < 36 had increased XOR activity and serum UA levels. Independent of insulin resistance and serum UA levels, multivariate logistic regression analysis revealed that plasma XOR activity was associated with the risk of hepatic steatosis as assessed by the L/S ratio and HSI. According to the findings of this study, plasma XOR activity is associated with hepatic steatosis independent of insulin resistance and serum UA levels.

## Introduction

Nonalcoholic fatty liver disease (NAFLD) is defined as the presence of fatty accumulation in the liver on imaging or histology, excluding fatty liver secondary to alcohol, drugs, or genetic disorders^1,2^. NAFLD is a major cause of liver disease, and its prevalence is reported to be increasing^1–3^; NAFLD has been reported to be a risk factor for not only hepatic disease-related mortality but also cardiovascular disease^4–7^.

Imaging techniques, such as abdominal ultrasonography, computed tomography (CT), and magnetic resonance imaging, are useful in evaluating hepatic steatosis. Abdominal ultrasound is the recommended screening test for NAFLD, but CT has also been used to evaluate hepatic steatosis. The CT value of the liver decreases with the degree of fat deposition, and by measuring the ratio of the CT values in the liver to those in the spleen [the liver-to-spleen (L/S) ratio], it is possible to calculate the fat content of the liver^8–12^. In addition to imaging tests, the hepatic steatosis index (HSI) has been useful as screening indices for hepatic steatosis^13^.

Metabolic syndrome and type 2 diabetes mellitus, which are associated with insulin resistance, are known to be risk factors for the development of NAFLD^14–16^. In addition, hyperuricemia has been reported to be a risk factor for metabolic syndrome and NAFLD^17,18^. Xanthine oxidoreductase (XOR) is an enzyme regulating the synthesis of uric acid (UA) and generation of reactive oxygen species (ROS)^19,20^. XOR activity is associated with insulin resistance^21^ and is elevated in metabolic syndrome and type 2 diabetes^22,23^. In addition, previous studies demonstrated that XOR activity was significantly increased in a mouse model of NAFLD and that fatty liver induced by a high-fat diet was suppressed by administration of XOR inhibitors^24,25^.

It is assumed that XOR activity is associated with hepatic steatosis in humans. However, in humans, XOR activity is extremely low compared with that in rodents; this makes accurate measurement difficult^26–28^. Therefore, a novel human plasma XOR activity assay has been developed using a combination of liquid chromatography (LC) and triple quadrupole mass spectrometry (TQMS) to detect [^13^C_2_, ^15^N_2_] UA using [^13^C_2_, ^15^N_2_] xanthine as a substrate^26–28^.

The aim of this study was to clarify the relationship between XOR activity accurately evaluated by this novel method and hepatic steatosis assessed by the L/S ratio and HSI in humans.

## Results

### Characteristics of the study participants

Results are given as the median (interquartile range), unless otherwise stated. The characteristics of the participants are presented in Table 1. A total of 223 subjects were included (142 females and 81 males). The age was 66 (53–73) years; the body mass index (BMI) was 23.1 (21.2–26.4) kg/m^2^; the abdominal circumference (AC) assessed using CT was 83.9 (77.0–91.8) cm; the subcutaneous fat area (SFA) was 162.7 (108.1–221.8) cm^2^; and the visceral fat area (VFA) was 86.1 (53.1–119.0) cm^2^. The XOR activity was 42.7 (25.3–78.6) pmol/h/mL; the serum UA was 5.3 (4.5–6.2) mg/dL; the urinary UA to creatinine ratio (UACR) was 0.46 (0.40–0.55); the L/S ratio was 1.3 (1.2–1.5); and the HSI was 32.6 (29.3–37.9).

**Table 1:** Participant characteristics.

N (female:male)	223 (142:81)
Age (years)	66 (53–73)
BMI (kg/m^2^)	23.1 (21.2–26.4)
Abdominal circumference (cm)	83.9 (77.0–90.8)
Subcutaneous fat area (cm^2^)	162.7 (108.1–221.8)
Visceral fat area (cm^2^)	86.1 (53.1–119.0)
HbA1c (%)	5.8 (5.6–6.2)
HOMA–R	1.4 (0.9–2.2)
T-Chol (mg/dL)	194.0 (174.0–215.0)
TG (mg/dL)	108.0 (77.0–154.5)
HDL-Chol (mg/dL)	57.0 (48.5–70.0)
XOR activity (pmol/h/mL)	42.7 (25.3–78.6)
UA (mg/dL)	5.3 (4.5–6.2)
UACR	0.46 (0.40–0.55)
L/S ratio	1.3 (1.2–1.5)
HSI	32.6 (29.3–37.9)
AST (U/L)	20.0 (16.0–25.0)
ALT (U/L)	18.0 (13.0–26.0)
γ-GTP	21.0 (15.8–32.0)
Hypertension	171 (76.7%)
Diabetes	51 (22.9%)
Dyslipidemia	145 (65.0%)

The results are presented as median (interquartile range). BMI, body mass index; HbA1c, hemoglobin A1c; HOMA-R, homeostasis model assessment ratio; T-Chol, total-cholesterol; TG, triglycerides; HDL-Chol, high-density lipoprotein-cholesterol; XOR, xanthine oxidoreductase; UA, uric acid; UACR, urine uric acid to creatinine ratio; L/S, liver-to-spleen; HSI, hepatic steatosis index; AST, aspartate transaminase; ALT, alanine transaminase: γ-GTP, γ-glutamyl transpeptidas.

### Differences in patient background categorized by the L/S ratio and HSI

According to the previous reports^11,12^, the participants were divided into three groups based on their L/S ratios, < 1.1, 1.1–1.296, and > 1.296, and each parameter was compared (Table 2). As the L/S ratio decreased, the proportion of males significantly increased (P = 0.004), as did BMI, AC, SFA, VFA, and HOMA-R (all P < 0.001). Furthermore, as the L/S ratio decreased, plasma XOR activity and serum UA levels significantly increased (P < 0.001 for both).

**Table 2:** Differences in clinical parameters based on liver-to-spleen (L/S) ratio.

L/S ratio Variables	> 1.296 (N = 126)	1.1–1.296 (N = 61)	< 1.1 (N = 36)	P for trend
Female:Male	90:36	35:26	17:19	0.004
Age (years)	68 (59–75)	60 (49–70)	61 (52–71)	0.001
BMI (kg/m^2^)	22.3 (20.7–24.4)	24.6 (22.1–27.8)	26.2 (24.3–29.5)	< 0.001
AC (cm)	80.9 (73.9–86.3)	87.4 (80.2–94.7)	88.1 (85.3–99.5)	< 0.001
SFA (cm^2^)	144.4 (102.1–195.9)	181.0 (113.4–232.1)	184.4 (128.9–270.3)	< 0.001
VFA (cm^2^)	76.9 (47.0–99.2)	88.2 (50.3–128.3)	119.0 (98.4–156.1)	< 0.001
HbA1c (%)	5.8 (5.5–6.0)	5.8 (5.5–6.3)	5.9 (5.7–6.8)	0.164
HOMA-R	1.2 (0.8–1.8)	1.7 (1.1–2.2)	2.6 (1.6–3.5)	< 0.001
T-Chol (mg/dL)	192.0 (175.0–215.8)	194.0 (171.0–211.0)	198.0 (172.0–216.3)	0.557
TG (mg/dL)	96.5 (71.3–144.3)	111.0 (80.0–150.0)	160.0 (120.0–211.0)	< 0.001
HDL–Chol (mg/dL)	61.0 (52.0–74.0)	57.0 (45.0–66.0)	49.0 (42.8–57.3)	< 0.001
XOR activity (pmol/h/mL)	32.2 (22.8–52.8)	59.1 (25.9–92.8)	82.4 (61.5–137.5)	< 0.001
UA (mg/dL)	5.0 (4.2–6.0)	5.5 (4.8–6.4)	5.6 (5.0–6.5)	< 0.001
UACR	0.47 (0.41–0.55)	0.45 (0.37–0.54)	0.46 (0.40–0.52)	0.101
HSI	30.7 (28.3–33.7)	34.5 (31.2–39.2)	38.1 (35.3–42.0)	< 0.001
AST (U/L)	19.0 (15.0–23.0)	20.0 (16.0–24.0)	26.0 (20.8–31.5)	< 0.001
ALT (U/L)	16.0 (12.0–19.8)	20.0 (13.0–30.0)	34.0 (24.8–42.0)	< 0.001
γ-GTP (U/L)	19.0 (14.0–25.8)	24.0 (16.5–32.0)	36.0 (25.5–68.5)	< 0.001
Hypertension	89	53	29	0.031
Diabetes	24	17	10	0.137
Dyslipidemia	81	37	27	0.523

Clinical parameters and the proportion of comorbidities among the three groups were examined using Jonckheere–Terpstra test or Cochran–Armitage test.

BMI, body mass index; AC, abdominal circumference; SFA, subcutaneous fat area; visceral fat area, VFA; HbA1c, hemoglobin A1c; HOMA-R, homeostasis model assessment ratio; T-Chol, total-cholesterol; TG, triglycerides; HDL-Chol, high-density lipoprotein-cholesterol; UA, uric acid; UACR, urine uric acid to creatinine ratio; HSI, hepatic steatosis index; AST, aspartate transaminase; ALT, alanine transaminase: γ-GTP, γ-glutamyl transpeptidas.

According to the previous method^13^, the participants were also divided into three groups based on their HSI, < 30.0, 30.0–36.0, and > 36.0, and each parameter was compared (Table 3). The proportion of males significantly increased with increasing HSI (P = 0.009), as did BMI, AC, SFA, VFA, and HOMA-R (all P < 0.001). Furthermore, with increasing HSI, plasma XOR activity and serum UA levels significantly increased (both P < 0.001).

**Table 3:** Differences in clinical parameters based on hepatic steatosis index (HSI).

HSI Variables	< 30.0 (N = 68)	30.0 – 36.0 (N = 84)	> 36.0 (N = 71)	P for trend
Female:male	51:17	53:31	38:33	0.009
Age (years)	70 (64–76)	67 (54–73)	56 (47–67)	< 0.001
BMI (kg/m^2^)	20.5 (18.9–21.5)	23.1 (22.3–24.6)	28.5 (26.1–31.6)	< 0.001
AC (cm)	74.2 (70.5–80.6)	84.0 (79.7–87.7)	94.6 (88.5–101.9)	< 0.001
SFA (cm^2^)	105.0 (75.9–145.5)	163.9 (122.6–208.7)	230.1 (180.7–336.1)	< 0.001
VFA (cm^2^)	57.5 (30.4–86.3)	82.1 (57.3–108.1)	123.2 (85.9–163.0)	< 0.001
HbA1c (%)	5.7 (5.6–5.9)	5.7 (5.5–6.2)	5.9 (5.6–7.0)	0.015
HOMA-R	0.9 (0.6–1.5)	1.3 (1.0–2.0)	2.2 (1.4–2.8)	< 0.001
T-Chol (mg/dL)	193.0 (176.5–214.3)	194.5 (172.8–216.3)	194.0 (173.5–214.5)	0.585
TG (mg/dL)	84.5 (62.5–118.0)	107.0 (78.5–151.3)	128.0 (101.0–210.0)	< 0.001
HDL–Chol (mg/dL)	66.0 (54.8–78.3)	58.5 (51.5–70.5)	49.0 (43.0–57.5)	< 0.001
XOR activity (pmol/h/mL)	27.1 (19.2–43.0)	39.7 (25.6–73.6)	79.2 (47.2–127.0)	< 0.001
UA (mg/dL)	5.0 (4.1–5.4)	5.1 (4.4–6.3)	5.8 (5.2–6.5)	< 0.001
UACR	0.47 (0.42–0.57)	0.46 (0.40–0.55)	0.46 (0.38–0.53)	0.125
L/S ratio	1.4 (1.3–1.5)	1.3 (1.2–1.5)	1.2 (1.0–1.3)	< 0.001
AST (U/L)	19.5 (16.0–22.0)	20.0 (16.0–23.0)	24.0 (18.0–28.0)	0.002
ALT (U/L)	13.0 (9.0–17.0)	18.0 (13.8–24.0)	30.0 (20.5–39.5)	< 0.001
γ-GTP (U/L)	16.0 (12.0–24.0)	21.0 (16.0–31.3)	26.0 (21.0–43.0)	< 0.001
Hypertension	46	65	60	0.019
Diabetes	8	21	22	0.007
Dyslipidemia	35	33	40	0.633

Clinical parameters and the proportion of comorbidities among the three groups were examined using Jonckheere–Terpstra test or Cochran–Armitage test.

BMI, body mass index; AC, abdominal circumference; SFA, subcutaneous fat area; visceral fat area, VFA; HbA1c, hemoglobin A1c; HOMA-R, homeostasis model assessment ratio; T-Chol, total-cholesterol; TG, triglycerides; HDL-Chol, high-density lipoprotein-cholesterol; XOR, xanthine oxidoreductase; UACR, urine uric acid to creatinine ratio; L/S, liver-to-spleen; AST, aspartate transaminase; ALT, alanine transaminase.

### Assessment of liver fibrosis progression

In patients with the L/S ratio of < 1.1, the NAFLD fibrosis score (NFS) and the Fibrosis-4 (FIB-4) index were calculated to predict the progression of liver fibrosis^1,29–32^. In these subjects, the NFS was −2.086 (−2.836 to −0.532) with an NFS of > 0.676 observed in only three subjects, while the FIB-4 index was 1.09 (0.74–1.56) with an FIB-4 index of ≥ 2.67 observed in only four subjects.

### Association of the XOR activity and UA levels with hepatic steatosis

**Figure 1:**
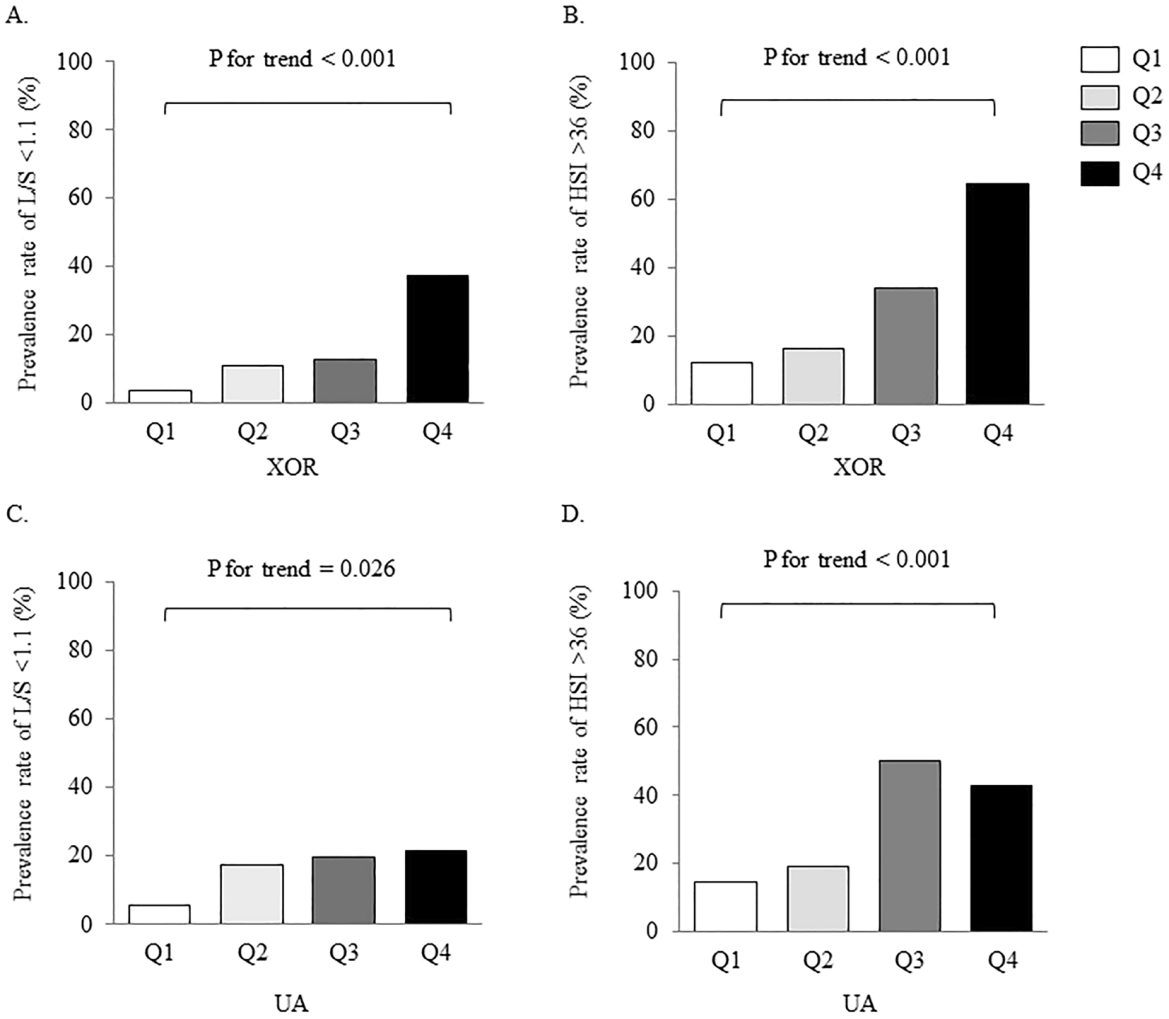
Comparisons of indices of hepatic steatosis among quadrants of xanthine oxidoreductase (XOR) activity or uric acid (UA) levels. (**A**) Plasma XOR activity and prevalence rate of liver-to-spleen (L/S) ratio < 1.1, (**B**) Serum UA levels and prevalence rate of L/S ratio < 1.1, (**C**) XOR and prevalence rate of hepatic steatosis index (HSI) > 36.0, (**D**) UA levels and prevalence rate of HSI > 36.0. The proportion of hepatic steatosis between quartiles was examined using the Cochran–Armitage test. Abbreviations: Q, quadrant.

Participants were divided into quartiles based on their XOR activity. The proportion of each XOR activity with an L/S ratio of < 1.1 and an HSI of > 36.0 is shown in Fig. 1. The proportions of subjects with the L/S ratio of < 1.1 and the HSI of > 36.0 significantly increased with increasing XOR activity (both P < 0.001). Serum UA levels were used to divide participants into quartiles. Similar to XOR activity, increasing UA levels increased the proportion of subjects with the L/S ratio of < 1.1 (P = 0.026) and the HSI of > 36.0 (P < 0.001).

An ordinal logistic regression analysis using the L/S ratio as the objective variable and UA levels as the explanatory variable revealed that UA levels had a crude odds ratio (OR) of 1.454 [95% confidence interval (CI): 1.159–1.824, P < 0.001] (Table 4). A similar analysis revealed that a crude OR for XOR activity per 10 pmol/h/mL of 1.080 (95% CI: 1.039–1.123, P < 0.001). Thus, both UA levels and XOR activity were associated with a lower L/S ratio. Next, since hepatic steatosis, hyperuricemia, and XOR activity were all associated with insulin resistance, we ran a logistic regression analysis in Model 1 with UA levels, XOR activity, and HOMA-R as explanatory variables. Results showed that XOR activity and HOMA-R were associated with a lower L/S ratio, with an OR for XOR activity per 10 pmol/h/mL of 1.052 (95% CI: 1.013–1.093, P = 0.009) and an OR for HOMA-R of 1.593 (95% CI: 1.230–2.062, P < 0.001), but UA levels were not, with an OR of 1.228. Furthermore, because NAFLD is known to be associated with obesity, hypertension, dyslipidemia, and diabetes mellitus^1,3,14^, we performed a logistic regression analysis in Model 3 that adjusted for age, sex, and the presence of hypertension, dyslipidemia, and diabetes mellitus. The results indicated that XOR activity was associated with a lower L/S ratio, with an OR for XOR activity per 10 pmol/h/mL of 1.047 (95% CI: 1.009–1.086, P = 0.016) independent of HOMA-R and serum UA levels.

**Table 4:** Logistic regression analyses of the factors associated with liver-to-spleen (L/S) ratio.

	L/S ratio							
			Model 1 (n = 216)		Model 2 (n = 216)		Model 3 (n = 216)	
	Crude OR (95% CI)	P	OR (95% CI)	P	OR (95% CI)	P	OR (95% CI)	P
UA	1.454 (1.159–1.824)	< 0.001	1.228 (0.960–1.570)	0.102	1.128 (0.875–1.454)	0.354	1.047 (0.800–1.370)	0.737
XOR activity per 10 pmol/h/mL	1.080 (1.039–1.123)	< 0.001	1.052 (1.013–1.093)	0.009	1.047 (1.009–1.086)	0.016	1.047 (1.009–1.086)	0.016
HOMA-R	1.836 (1.437–2.347)	< 0.001	1.593 (1.230–2.062)	< 0.001	1.411 (1.070–1.860)	0.015	1.407 (1.050–1.884)	0.022
BMI	1.190 (1.117–1.268)	< 0.001			1.102 (1.022–1.188)	0.012	1.087 (1.006–1.174)	0.034

In model 1, an ordinal logistic regression analysis was performed with L/S ratio as the objective variable and serum uric acid (UA) levels, plasma xanthine oxidoreductase (XOR) activity, and homeostasis model assessment ratio (HOMA-R) as explanatory variables. In Model 2, body mass index (BMI) was added as an explanatory variable. In Model 3, logistic regression analysis was performed adjusting for age, gender, and presence of hypertension, dyslipidemia, and diabetes mellitus.

OR, odds ratio; CI, confidence interval.

Table 5 shows the results of HSI as a dependent factor. Independent of HOMA-R and serum UA levels, XOR activity was associated with increased HSI, with an OR for XOR activity per 10 pmol/h/mL of 1.158 (95% CI: 1.085–1.237, P < 0.001).

**Table 5:** Logistic regression analyses of the factors associated with hepatic steatosis index (HSI).

	HSI			
			Model 1 (n = 216)	
	Crude OR (95% CI)	P	OR (95% CI)	P
UA	1.583 (1.276–1.964)	< 0.001	1.298 (1.018–1.654)	0.035
XOR activity per 10 pmol/h/mL	1.227 (1.144–1.315)	< 0.001	1.158 (1.085–1.237)	< 0.001
HOMA-R	2.413 (1.795–3.245)	< 0.001	1.878 (1.379–2.559)	< 0.001

In model 1, an ordinal logistic regression analysis was performed with HSI as the objective variable and serum uric acid (UA) levels, plasma xanthine oxidoreductase (XOR) activity, and homeostasis model assessment ratio (HOMA-R) as explanatory variables.

OR, odds ratio; CI, confidence interval.

## Discussion

XOR is an enzyme that controls the synthesis of UA. XOR has also been reported to cause vascular endothelial dysfunction through adipogenesis and ROS production^33–36^. Furthermore, XOR activity has been reported to induce hepatic steatosis via ROS production and activation of the c-Jun *N*-terminal kinase^37^. In animal experiments, it has been reported that inhibiting XOR can suppress hepatic steatosis^24,25^. Therefore, it is assumed that XOR activity and hepatic steatosis are related in humans; however, measuring XOR activity in humans has proven difficult. XOR activity was accurately measured in this study using LC/TQMS, and it was found that XOR activity is associated with hepatic steatosis in humans.

UA itself has been reported to induce hepatic lipid accumulation by inducing mitochondrial oxidative stress and insulin resistance^38,39^. In fact, it has been reported that hyperuricemia is associated with the development and progression of NAFLD^17,18,40^. According to the findings of this study, plasma XOR activity was more useful than serum UA levels as an explanatory variable for the lower L/S ratio. However, because this study primarily aimed to investigate the relationship between XOR activity and hepatic steatosis, we excluded patients taking XOR inhibitors, which could interfere with measuring plasma XOR activity. In fact, there were only 17 (7.6%) patients with untreated hyperuricemia (serum UA ≥ 7.0 mg/dL) in this study. Therefore, further studies including patients with hyperuricemia may be necessary to clarify the relationship between UA levels or XOR activity and hepatic steatosis.

Insulin resistance causes hepatic steatosis via various mechanisms, including promoting the transfer of free fatty acids from adipose tissue to the liver, and is a major risk factor for the development of NAFLD^41–44^. Furthermore, insulin resistance raises the risk of hyperuricemia by increasing not only XOR activity but also UA reabsorption^21,45^. On the other hand, a study using mice with genetically disrupted hepatocyte XOR found that suppressing hyperuricemia through hepatic XOR inhibition did not improve systemic metabolic abnormalities, including insulin resistance^46^. However, that paper did not directly evaluate the relationship between XOR activity or UA levels and hepatic steatosis^46^. It is important to note that the findings of this study show that XOR activity is associated with hepatic steatosis independent of insulin resistance. Long-term prospective studies are needed, however, to better understand the relationship between XOR activity and hepatic steatosis.

In this study, the HSI and L/S ratios were also used to assess hepatic steatosis. According to a case–control study conducted in Korea, NAFLD can be excluded with a negative likelihood ratio of 0.186 for HSI < 30.0 and detected with a positive likelihood ratio of 6.069 for HSI > 36.0^13^. Furthermore, in a study of Western subjects, the L/S ratio was found to have a sensitivity of 93% for detecting moderate to severe fatty liver^9^. A study using liver transplant donors in Japan found that the L/S ratio > 1.1 was sufficient to rule out moderate or severe fatty liver^12^. Furthermore, when liver biopsies were performed to histologically evaluate the liver and compared with the L/S ratio, it was reported that the L/S ratio cutoff value for detecting clinically problematic fatty liver was 1.1, and the L/S ratio assuming the absence of hepatic steatosis was higher than 1.296^11^. However, it has been reported that using the L/S ratio to assess mild fatty liver is difficult^9^. Therefore, further investigation, including echography and liver biopsy, is considered necessary to accurately evaluate hepatic steatosis.

In this study, we used the NFS and the FIB-4 index to evaluate the progression of hepatic fibrosis^1,29–32^, but only three patients with an L/S ratio < 1.1 had a high NFS, and only four patients had a high FIB-4 index, and no significant relationship between hepatic fibrosis and XOR activity was found. Due to the high invasiveness of liver biopsy, it was not performed in this study. However, a prospective study with liver biopsy is required to thoroughly investigate the relationship between NAFLD onset and progression and XOR activity.

The limitation of this study is that it was a single-center, cross-sectional analysis. In this study, we used the L/S ratio and HSI as indices of hepatic steatosis and NFS and the FIB-4 index as indices of hepatic fibrosis progression; however, a liver biopsy is still necessary to diagnose fatty liver, and further studies are required. Additionally, long-term prospective studies including patients with hyperuricemia are needed to better understand the relationship between XOR activity or serum UA levels and hepatic steatosis.

In conclusion, the findings of this study show that plasma XOR activity is associated with hepatic steatosis independent of insulin resistance and serum UA levels. Thus, XOR activity may be involved in hepatic steatosis in humans.

## Methods

### Study design and participants.

This cross-sectional analysis was conducted as part of the Hyogo Sleep Cardio-Autonomic Atherosclerosis (HSCAA) study^47,48^. The HSCAA study is a single-center cohort study that aims to investigate the interrelationships among sleep disorders, autonomic neuropathy, metabolic diseases, and atherosclerotic diseases^47,48^. The HSCAA study included patients aged 20 years and older with one or more cardiovascular risk factors (obesity, smoking, cardiovascular event history, hypertension, dyslipidemia, diabetes mellitus, chronic kidney disease) and being treated at the Hyogo College of Medicine Hospital.

Since we started XOR measurements from 2018 for the subjects who were registered or followed in the HSCAA study, this cross-sectional study included 310 patients, from January 2018 to July 2021, who consented to abdominal CT examinations. In the end, 223 patients were analyzed in the present study after excluding 87 with alcoholic habits (> 30 g/day for males and > 20 g/day for females), autoimmune hepatitis, viral hepatitis, or under treatment with XOR inhibitors.

The HSCAA study has been approved by the Ethics Committee of Hyogo College of Medicine Hospital (Approval No. 2351). Written informed consent was obtained from all subjects and the study was conducted in full accordance with the Declaration of Helsinki. The present study protocol was approved by the Ethics Committee of Hyogo College of Medicine Hospital (Approval No. 3601) and performed with an opt-out option, as explained in instructions posted on the website of the hospital. All methods in our study were performed in accordance with the relevant guidelines and regulations.

### Visceral fat area and subcutaneous fat area

CT was performed using SIEMENS SOMATOM Definition AS + or SOMATOM Definition H (Siemens Healthcare GmbH, Erlangen, Germany) with 10 mm slices. We evaluated the visceral fat area (VFA), subcutaneous fat area (SFA), and waist circumference using Ziostation 2 (AMIN Ltd., Tokyo, Japan). The AC was measured at the umbilical height.

### Hepatic steatosis and liver fibrosis

The L/S ratio and HSI were used to evaluate hepatic steatosis. Hepatic and splenic attenuation values were measured on non-contrast-CT scans using four circular region-of-interest (ROI) cursors in the liver and two in the spleen. In the liver, four ROIs were located in each of the right lobe and the left lobe. All measurements were manually obtained in regions of uniform parenchymal attenuation, with care being taken to avoid vessels, artifacts, and other areas that might have spuriously increased or decreased measurements. The calculation of the L/S ratio was as follow: L/S ratio = (Average attenuation value of the liver) / (Average attenuation value of the spleen)^9–12^. HSI was calculated from ALT, AST, BMI, sex, and the presence of diabetes mellitus^13^.

In addition, the NAFLD fibrosis score (NFS) and the Fibrosis-4 (FIB-4) index were calculated to predict the progression of liver fibrosis in patients with the L/S ratio of < 1.1^1,29–32^. NFS was calculated from age, BMI, AST, ALT, the presence of glucose intolerance, platelet count, and albumin^31,32^. It has been reported that by applying the high cutoff score (NFS > 0.676), the presence of advanced fibrosis could be diagnosed with high accuracy^31,32^. The FIB-4 index was calculated from age, AST, ALT, and platelet count. It has been reported that its cutoff value < 1.45 can exclude hepatic fibrosis, and its cutoff value > 2.67 can predict hepatic fibrosis^30^.

### Plasma XOR activity measurement

The assay protocol of XOR activity in humans was reported previously^26–28^. In brief, 100 μL of plasma samples (purified by Sephadex G25 resin) were mixed with a Tris buffer (pH 8.5) containing [^13^C_2_,^15^N_2_] xanthine as a substrate, NAD+, and [^13^C_3_,^15^N_3_] UA as an internal standard. The mixtures were incubated at 37 °C for 90 min, mixed with 500 µL of methanol, and centrifuged at 2000× *g* for 15 min at 4 °C. The supernatants were transferred to new tubes and dried using a centrifugal evaporator. The residues were reconstituted with 150 μL of distilled water, filtered through an ultrafiltration membrane, and measured using LC/TQMS. LC/TQMS comprised a Nano Space SI-2 LC system (Shiseido Co., Ltd., Tokyo, Japan) and a TSQ Triple Quadrupole LC–MS system (ThermoFisher Scientific GmbH, Bremen, Germany) equipped with an ESI interface. Calibration standard samples of [^13^C_2_,^15^N_2_] UA were also measured, and the amounts of production were quantitated from the calibration curve. XOR activities were expressed in pmol/mL/h^26–28^.

### Other parameters

At the same time as that for the CT scan, blood samples were taken for AST, ALT, UA, fasting blood glucose, immunoreactive insulin, total cholesterol (T-Chol), high-density lipoprotein cholesterol (HDL-Chol), and TG. In addition, serum UA levels were measured using the uricase/peroxidase technique with an autoanalyzer (Pureauto S UA Sekisui Medical, Ltd., Tokyo, Japan). Height, weight, and blood pressure were also measured.

Type 2 diabetes was diagnosed based on results showing fasting plasma glucose ≥ 126 mg/dL, causal plasma glucose ≥ 200 mg/dL, or 2-h plasma glucose ≥ 200 mg/dL during a 75-g oral glucose tolerance test, or previous therapy for diabetes^49^. Hypertension was defined as systolic blood pressure ≥ 140 mmHg, diastolic blood pressure ≥ 90 mmHg, or taking treatment for hypertension. We defined dyslipidemia as the presence of LDL-C ≥ 140 mg/dL, HDL-C ≤ 40 mg/dL, TG level ≥ 150 mg/dL, or taking treatment for dyslipidemia.

### Statistical analysis

The results were presented as median (interquartile range), unless otherwise stated. We used the Jonckheere-Terpstra test to compare the trend of data between three or more groups. The Cochran–Armitage test was used for the trend of the ratio between three or more groups.

Hepatic steatosis was graded as follows: with hepatic steatosis (L/S ratio < 1.1)^11,12^, without hepatic steatosis (L/S ratio > 1.296), and intermediate (L/S ratio = 1.1–1.296)^11^. In model 1, an ordinal logistic regression analysis was performed with L/S ratio as the objective variable and serum UA levels, plasma XOR activity, and HOMA-R as explanatory variables. In Model 2, BMI was added as an explanatory variable. In Model 3, we used an ordinal logistic regression analysis, and the L/S ratio was used as the objective variable; UA, XOR activity, and the HOMA-R were used as the explanatory variables, adjusted for age, sex, and components for Japanese diagnostic criteria of metabolic syndrome (AC, blood pressure, plasma glucose, HDL, and TG).

HSI of > 36.0, < 30, and 30 − 36 were defined as high, low, and intermediate, respectively, based on the previous report^13^. Then, an ordinal logistic regression analysis was performed with HSI as the objective variables and UA, XOR, and HOMA-R as explanatory variables.

Statistical analyses were conducted using the BellCurve software version 2.15 (Social Survey Research Information Co., Ltd., Tokyo, Japan), with P < 0.05 indicating statistical significance.

## Data Availability

The datasets generated during and/or analysed during the current study are available from the corresponding author on reasonable request.
